# lncRNA NEAT1 regulates CYP1A2 and influences steroid-induced necrosis

**DOI:** 10.1515/biol-2021-0097

**Published:** 2021-09-13

**Authors:** Yongfang Zhou, Fei Zhang, Fengyang Xu, Qiang Wang, Jianhua Wu, Wuxun Peng, Wentao Dong

**Affiliations:** Department of Trauma Orthopedics, The Affliated Hospital of Guizhou Medical University, Guiyang, Guizhou 550004, China; School of Clinical Medicine, Guizhou Medical University, Guiyang, Guizhou 550004, China

**Keywords:** SNFH, lncRNA NEAT1, hBMSCs, osteogenic differentiation

## Abstract

The main cause of steroid-induced necrosis of femoral head (SNFH) is excessive glucocorticoid (GC) intake. The aim of this article was to investigate the role of lncRNA NEAT1 as a molecular sponge to adsorb miR-23b-3p and regulate CYP1A2 in SNFH. Fluorescence *in situ* hybridization was used to localize lncRNA NEAT1. Human bone marrow mesenchymal stem cells (hBMSCs) were collected from patients with SNFH. The expression of lncRNA NEAT1, miR-23b-3p and CYP1A2 in hBMSCs were intervened. Compared to the control group, the lncRNA NEAT1 and CYP1A2 expression in the SNFH group was increased, while miR-23b-3p expression was decreased. GCs could inhibit the osteogenic differentiation of hBMSCs and upregulate the expression of lncRNA NEAT1. Knockdown of lncRNA NEAT1 could promote the proliferation and osteogenic differentiation of hBMSCs in the SNFH group. Overexpression of miR-23b-3p could partially counteract the effect of lncRNA NEAT1 on hBMSCs. CYP1A2 was confirmed to be a target of miR-23b-3p. Overexpression of CYP1A2 could partially rescue the effect of miR-23b-3p overexpression on hBMSCs. In conclusion, lncRNA NEAT1 as a ceRNA can adsorb miR-23b-3p and promote the expression of CYP1A2, which then inhibits the osteogenic differentiation of hBMSCs and promotes the progress of SNFH.

## 1 Introduction

Osteonecrosis of the femoral head (ONFH) is also known as avascular necrosis of the femoral head. As the name suggests, it refers to the structural changes caused by the damage to the blood supply of the femoral head. It is one of the common diseases of lower limb bone disability in young adults [1]. According to the different causes of the disease, ONFH is divided into traumatic and non-traumatic femoral head necrosis. Most non-traumatic femoral head necrosis is caused by glucocorticoids (GCs) [2]. In recent years, people’s understanding of steroid-induced necrosis of femoral head (SNFH) has increased. However, the pathogenesis of SNFH is not very clear. Many studies have proposed the theory of thrombosis, apoptosis and weakened osteogenic differentiation ability [3,4]. Apoptosis is autonomous and programmed cell death, which plays an important role in the progress of many diseases [5].

Human bone marrow mesenchymal stem cells (hBMSCs) are tissue stem cells with multi-directional differentiation potential, which can differentiate into bone, fat, myocardial and other cells and play a role in the treatment of a variety of diseases [6]. In this study, we explored the function and mechanism of osteoblast differentiation of hBMSCs.

Long non-coding RNAs (lncRNAs) are a hot topic in recent years. In the past few years, lncRNAs have been shown to be involved in various pathological processes and have a profound impact on diseases. Many lncRNAs have been identified to play a functional role in SNFH. Wang et al. used gene chips and bioinformatics methods to study the expression of lncRNAs in bone marrow mesenchymal stem cells (BMSCs) of SNFH patients. They found that the expression of 1878 lncRNAs increased, and the expression of 1878 lncRNAs decreased, suggesting that many lncRNAs are involved in the pathogenesis of SNFH [7]. Xiang et al. confirmed that lncRNA RP11-154D6 was downregulated in SNFH, and it could promote osteogenic differentiation of BMSCs, playing an active role in SNFH [8]. According to previous studies, lncRNA NEAT1 has many functions, such as playing a tumor-promoting role in colorectal cancer and increasing the tolerance of nasopharyngeal carcinoma to histone-deacetylase inhibition [9,10]. lncRNA NEAT1 not only plays a role in cancer but also promotes renal fibrosis [11]. The effect of lncRNA NEAT1 on bone has also been studied. Overexpression of NEAT1 can stimulate the production of osteoclasts and reduce bone mass in mice [12]. NEAT1 is also highly expressed in malignant bone tumors and participates in osteosarcoma formation [13]. However, the mechanism of lncRNA NEAT1 in SNFH has not been discussed yet.

microRNAs (miRNAs) are a class of small RNA molecules without coding function. miRNAs can be competitively adsorbed by lncRNA, and thus their biological characteristics are inhibited. miRNAs can also target messenger RNA (mRNA) to affect its corresponding protein expression and form a lncRNA–miRNA–mRNA regulatory network [14]. It has been proved that miR-23b-3p can be regulated by lncRNA NEAT1, and miR-23b-3p can inhibit the progress of SNFH by inhibiting ZNF667 [15]. Wei et al. found that CYP1A2 served as a downstream target of miR-23b-3p and was associated with the risk genes of SNFH, and upregulation of miR-320 could reduce the risk of SNFH by inhibiting CYP1A2 [16].

In this study, GCs were used to induce hBMSCs to form an *in vitro* injury model. The effect of lncRNA NEAT1 on the hBMSC injury model was observed to explore the biological function of lncRNA NEAT1 in SNFH and the pathogenesis of SNFH, hoping to provide a new plan for the treatment of SNFH.

## 2 Materials and methods

### 2.1 Sample collection

Femoral head samples of 25 patients who received total hip arthroplasty in our hospital (The Affliated Hospital of Guizhou Medical University) from 2017 to 2019 were collected. The study enrolled 15 male and 10 female patients with a mean age of 42.2 ± 13.5 years. All enrolled patients had no history of smoking, drinking, chronic diseases or infectious diseases. The patients in the experimental group were those with steroid-induced necrosis of the femoral head: their GC intake threshold was more than 1,800 mg, or they had been receiving long-term steroid treatment for 4 weeks or more. The patients in the control group were those with femoral head necrosis secondary to senile femoral neck fractures, and they had no GC treatment history. Patients were diagnosed with femoral head necrosis by imaging examination before the operation and diagnosed as femoral head necrosis by pathological examination postoperatively.

**Informed consent:** Informed consent was obtained from all the individuals included in this study.**Ethical approval:** The research related to human use has complied with all relevant national regulations, institutional policies and is in accordance with the tenets of the Helsinki Declaration, and has been approved by the authors’ institutional review board or equivalent committee (approval no. 2001074).

### 2.2 Cell isolation and culture

HBMSCs were extracted during total hip arthroplasty. The specific operation is as follows. During the operation, 5 mL of bone marrow was extracted with a sterile syringe from the bone marrow cavity of the proximal femoral head, and the bone marrow fluid was added into the centrifuge tube to prepare a cell suspension. Lymphocyte separation solution (abs930; Shanghai Absin Biotechnology Co., Ltd., China) was added to the suspension and centrifuged at 2,000 rpm for 30 min. Monocytes from the white layer were collected and cultured in Dulbecco’s Modified Eagle Medium (10567014; Thermo Fisher Scientific, USA) at 37°C in a 5% CO_2_ incubator. The medium was changed every 3 days. Flow cytometry was used to detect the surface antigens expressed by hBMSCs, such as CD90, CD34 and CD45. Strong positive expression of CD90 and negative expression of CD34 and CD45 indicated successful isolation of hBMSCs [17].

### 2.3 Grouping and transfection

The overexpression plasmids, siRNAs and miR-23b-3p mimics were constructed by Shanghai GenePharma Co., Ltd. (China). The groups were as follows: si-NC group (transfected with negative control of knockdown gene), si-NEAT1 group (transfected with NEAT1 siRNA), OE-NC group (transfected with negative control of overexpression plasmid), OE-NEAT1 group (transfected with NEAT1 overexpression plasmid), OE-NEAT1 + miR-NC group (transfected with NEAT1 overexpression plasmid and injected with miRNA negative control), OE-NEAT1 + miR-23b-3p mimic group (transfected with NEAT1 overexpression plasmid and injected with miR-23b-3p mimic), miR-NC group (injected with miRNA negative control), miR-23b-3p mimic group (injected with miR-23b-3p mimic), miR-23b-3p mimic + NC group (injected with miR-23b-3p mimic and transfected with negative control of overexpression plasmid) and miR-23b-3p mimic + OE-CYP1A2 group (injected with miR-23b-3p mimic and transfected with CYP1A2 overexpression plasmid). The plasmids and siRNAs were transfected into hBMSCs by Lipofectamine 3000 kit (L3000001; Thermo Fisher Scientific, USA). Forty-eight hours after transfection, the experiments below were carried out.

### 2.4 Fluorescence *in situ* hybridization (FISH)

To localize lncRNA NEAT1, an FISH kit (C002; Shanghai Gefan Biotechnology Co., Ltd., China) was used. In short, the sample sections were incubated with a pre-hybridization solution at 65°C for 1 h. lncRNA NEAT1 probe was added to cover the sections, which were then incubated at 65°C for 48 h. After washing with saline-sodium citrate, an fluorescein isothiocyanate-labeled antibody was added to cover the sections. The sections were incubated in the darkroom for 30 min and then were washed with phosphate buffered saline (PBS). Finally, the sections were stained with DAPI and sealed with nail polish. The sections were observed under an inverted fluorescence microscope (DYF-880; Shanghai Dianying Optical Instrument Co., Ltd., China). Refer to the kit manual for detailed steps.

### 2.5 Dual-luciferase reporter assay

The binding site between lncRNA NEAT1 and miR-23b-3p was predicted using the Starbase database (http://starbase.sysu.edu.cn). The predicted lncRNA NEAT1 sequence was inserted into a pGL3 vector (HG-VQP0121; Changsha Aonuo Gene Technology Co., Ltd., China) to construct pGL3-NEAT1-WT and pGL3-NEAT1-MUT reporter vectors. The two reporter vectors were co-transfected with miR-23b-3p mimic and miR-23b-3p NC, respectively, into hBMSCs using Lipofectamine 3000 transfection reagent (L3000001; Thermo Fisher Scientific, USA). Forty-eight hours after transfection, the transfected cells were lysed with lysis buffer and centrifuged. The supernatant was used to detect luciferase activity with a dual-luciferase kit (11402ES60; Shanghai Yeasen Biotechnology Co., Ltd., China). The miR-23b-3p and CYP1A2 sequences were found on NCBI website, and the RNA22 database (https://cm.jefferson.edu/rna22/) was used to predict the binding site of miR-23b-3p and CYP1A2. The dual-luciferase reporter assay was designed with the same method above to verify the interaction between miR-23b-3p and CYP1A2.

### 2.6 qRT-PCR

TRIzol reagent (15596026; Thermo Fisher Scientific, USA) was used to extract RNA from tissues, and the quality of RNA was determined. RNA was used as the template and reverse transcribed into cDNA, which was completed by the reverse transcription kit (11141ES10; Shanghai Yeasen Biotechnology Co., Ltd., China). Real-time PCR was completed by the qRT-PCR kit (A15299; Thermo Fisher Scientific, USA) and the qRT-PCR system (7,500; Applied Biosystems, USA). The above operation steps were carried out according to the manufacturer’s instructions. GAPDH and U6 were chosen as the internal reference, and the results were calculated and analyzed according to the 2^−ΔΔCt^ method. The primers were as follows: lncRNA NEAT1 primers (forward, 5′-TGGCTAGCTCAGGGCTTCAG-3′; reverse, 5′-TCTCCTTGCCAAGCTTCCTTC-3′); miR-23b-3p primers (forward, 5′-ACACTCCAGCTCCCATCACATTGCCAGGGAT-3; reverse, 5′-CTCAACTGGTGTCGTGGAGCGAGGTGGTAAT-3′); CYP1A2 primers (forward, 5′-TAGCTCAGCTAGCTCGA-3; reverse, 5′-CTAGCTACGCGCTCGCTCG-3′); GAPDH primers (forward, 5′-TGAACGGGAAGCTCACTGG-3′; reverse, 5′-TCCACCACCCTGTTGCTGTA-3′); and U6 primers (forward, 5′-CTCGCTTCGGCAGCACA-3′; reverse, 5′-AACGCTTCACGAATTTGCGT-3′).

### 2.7 Alizarin red staining (ARS)

The hBMSCs from patients with femoral head necrosis secondary to senile femoral neck fractures were selected as the experimental subjects. Cells were seeded on 6-well plates. When cells reached 60–70% confluence, the medium was changed with the osteogenic differentiation medium to induce differentiation. The osteogenic differentiation medium was freshly prepared before use, which contained basic medium, 10% fetal bovine serum, 10 nmol/L dexamethasone (D8040; Beijing Solarbio Co., Ltd., China), 10 nmol/L ascorbic acid (A8100; Beijing Solarbio Co., Ltd., China), 50 mol/mL of glycerol phosphatide (Sigma-Aldrich, USA), 1% penicillin–streptomycin and 1% HEPES (H8090; Beijing Solarbio Co., Ltd., China). After 21 days of osteogenic differentiation, hBMSCs in the 6-well plate were washed with PBS and fixed with neutral formalin for 30 min. The fixation fluid was discarded, and the cells were rewashed with PBS. One milliliter of ARS agent (G8550; Beijing Solarbio Co., Ltd., China) was added to the wells for staining for 5 min. Then cells were washed and dried. The staining effect was observed under a microscope (DYF-880; Shanghai Dianying Optical Instrument Co., Ltd., China).

### 2.8 Alkaline phosphatase (ALP) staining

First, hBMSCs were treated according to the ARS staining method mentioned above. Then, hBMSCs cultured for 21 days were stained with an ALP staining agent (G1480; Beijing Solarbio Co., Ltd., China). Detailed steps were as follows. Cells were washed with PBS and fixed with 70% ethanol. The fixation solution was discarded. Cells were rewashed with PBS, stained with the ALP staining agent for 3 h and washed with distilled water. The staining effect was observed under a microscope.

### 2.9 CCK8 assay

Cell viability was detected by the CCK8 kit (HB-CCK8-1; Shanghai Hanheng Biotechnology Co., Ltd., China). The cell suspension was prepared, seeded in 96-well plates and cultured at 37°C in an incubator containing 5% CO_2_. Ten microliters of CCK8 solution were added to each well. Cells were then cultured in the incubator for an hour. The absorbance at 450 nm of each well was detected by the Varioska LUX multimode microplate reader (Thermo Fisher, USA), three times per well.

### 2.10 Western blot

Total protein was extracted from cells using the ProteoPrep^®^ Total Extraction Sample Kit (PROTTOT; Sigma-Aldrich, USA). Samples were heated to denature proteins and stored at −20°C. Twenty microliters of each protein sample were taken for the immunoblotting experiment. Proteins were separated by sodium dodecyl-sulfate polyacrylamide gel electrophoresis and transferred to a polyvinylidene fluoride membrane. The membrane was blocked with non-fat milk for 3 h. The primary antibodies at 1:1,000 dilution (CYP1A2 antibody: ab151728, Abcam, UK; GAPDH antibody: ab8245, Abcam, UK) were, respectively, added to the membrane and incubated overnight. After washing with TBST, ALP-conjugated secondary antibody (1:8,000, ab133470; Abcam, UK) was added to the membrane and incubated for an hour. Finally, the membrane was washed with TBST, and Novex AP chemiluminescent substrate (BioMag, Spain) was added to detect the signal. The gray scale values of the bands were analyzed.

### 2.11 Statistical analysis

All experiments were repeated three times. Data in this study were expressed as mean ± standard deviation. Comparison between the two groups was conducted by the *t*-test. Comparison of more than two groups was conducted by the one-way analysis of variance. SPSS 23.0 software was used to analyze the data. Figures and tables were made using the GraphPad Prism software. The correlation between two different variables was conducted by the Pearson correlation method. Receiver operating characteristic (ROC) curve was used to analyze the diagnostic value of lncRNA NEAT1 for SNFH. *P* < 0.05 was considered statistically significant.

## 3 Results

### 3.1 GCs inhibit the osteogenic differentiation of hBMSCs and upregulate lncRNA NEAT1 expression

**Figure 1:**
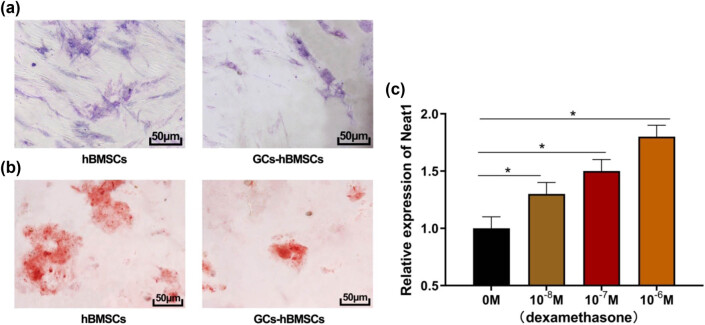
Effect of GCs on osteogenic differentiation of HBMSCs and lncRNA NEAT1 expression. (a) ALP staining (200×); (b) ARS staining (200×); (c) lncRNA NEAT1 expression of HBMSCs treated with dexamethasone at different concentrations. Compared with the control group, ^*^ *P* < 0.05. GCs: glucocorticoids; HBMSCs: human bone marrow mesenchymal stem cells; ALP: alkaline phosphatase; ARS: alizarin red staining.

On the 7th and 14th days of osteoinduction, hBMSCs in each group were stained for ALP and ARS. The results showed that compared to the control group, the ALP staining of hBMSCs induced by dexamethasone was lighter (Figure 1a), and the calcium nodules stained by ARS were fewer (Figure 1b), indicating the osteogenic differentiation ability of hBMSCs in the dexamethasone-induced group was weaker than that of the control group. Then 10^−8^, 10^−7^, and 10^−6^ M dexamethasone were added to the control group. The qRT-PCR results showed that the expression of lncRNA Neat1 increased as the concentration of dexamethasone increased (Figure 1c, all *P* < 0.05). The above results indicate that GCs can inhibit the osteogenic differentiation of hBMSCs and upregulate lncRNA NEAT1 expression.

### 3.2 miR-23b-3p is the target gene of lncRNA NEAT1

**Figure 2:**
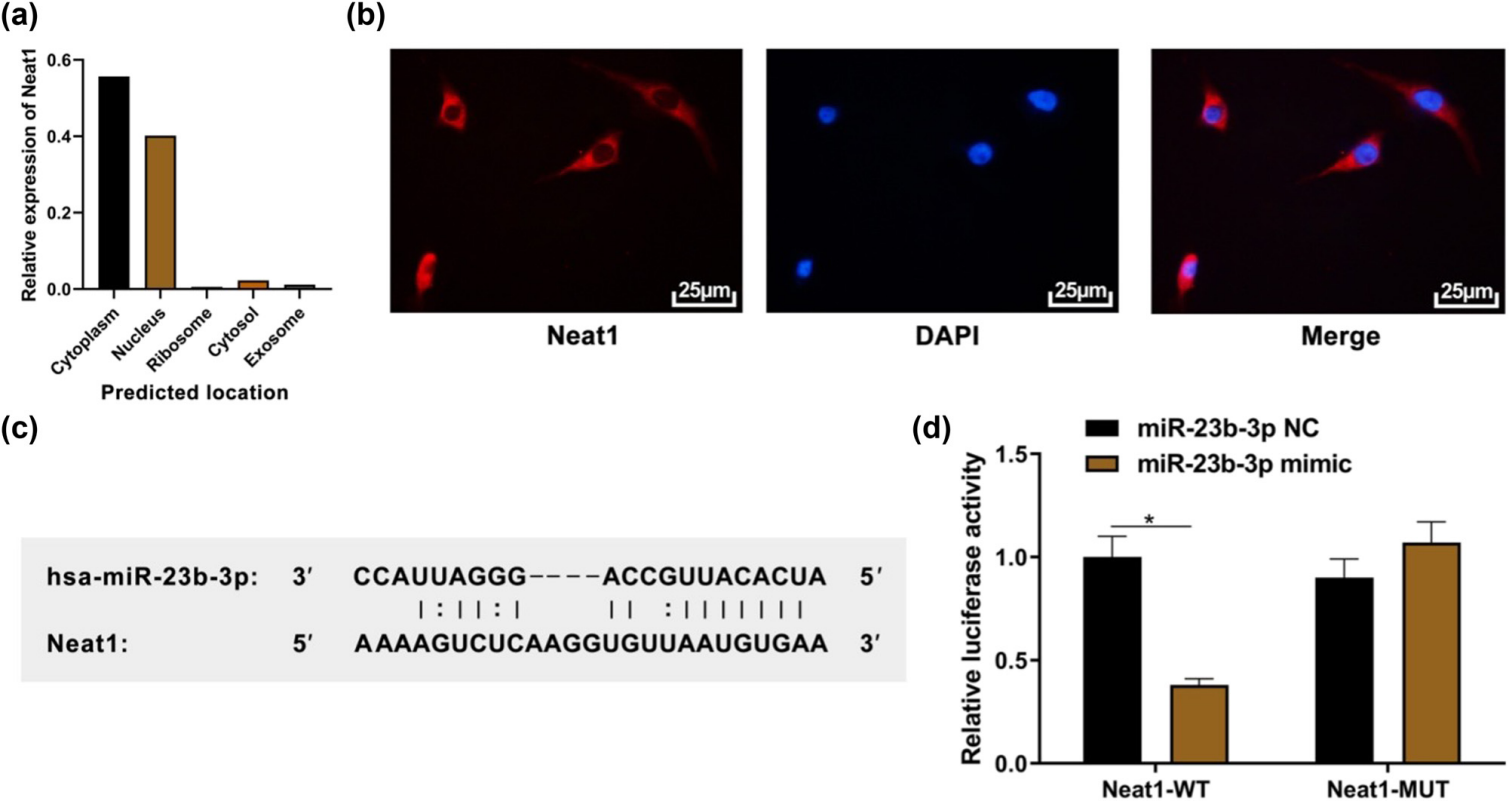
miR-23b-3p is the target gene of lncRNA NEAT1. (a) Subcellular location of lncRNA NEAT1 predicted by lncLocator website; (b) FISH showed that lncRNA NEAT1 was highly expressed in cytoplasm (400×); (c) the binding site of lncRNA NEAT1 and miR-23b-3p predicted by Starbase website; (d) dual luciferase reporter assay showed a targeting relationship between miR-23b-3p and lncRNA NEAT1. Compared with the control group, ^*^ *P* < 0.05.

lncRNA NEAT1 was found to be expressed in the cytoplasm through the lncLocator website (Figure 2a). Therefore, FISH was carried out to locate lncRNA NEAT1. The result showed that the expression of lncRNA NEAT1 in the cytoplasm was more than that in the nucleus (Figure 2b). The Starbase website predicted that lncRNA NEAT1 had a binding site with miR-23b-3p (Figure 2c), so the predicted sequence was used for dual-luciferase reporter assay. The result showed that compared to the control group, the luciferase activity of miR-23b-3p mimic and NEAT1-WT co-transfected cells was lower (Figure 2d, *P* < 0.05), while the luciferase activity of miR-23b-3p mimic and NEAT1-MUT co-transfected cells showed no significant difference (Figure 2d, *P* > 0.05). The above results indicate that lncRNA NEAT1 can act as ceRNA, which directly adsorb miR-23b-3p and react with it.

### 3.3 Increased expression of lncRNA NEAT1 and decreased expression of miR-23b-3p in SNFH

**Figure 3:**
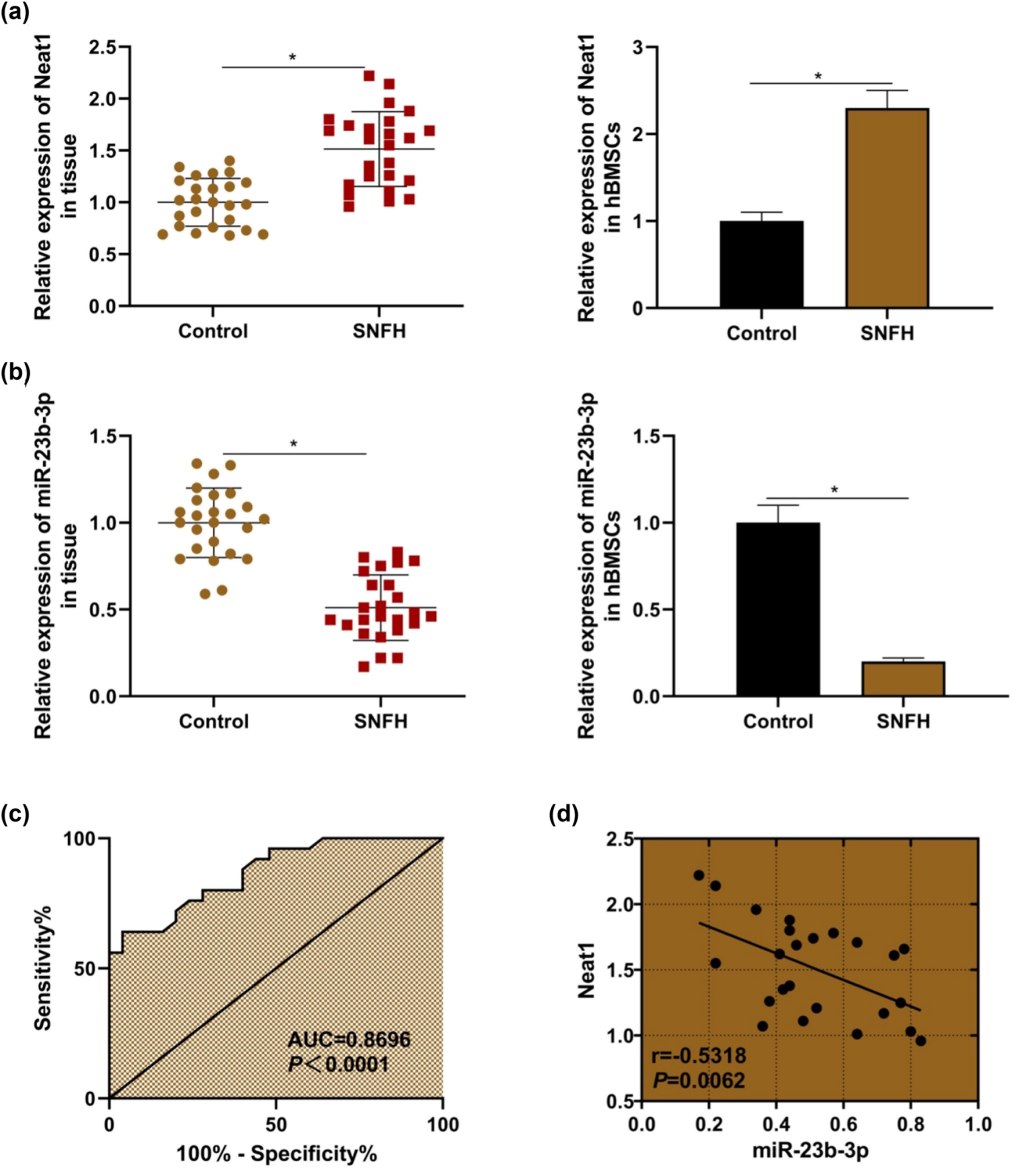
Increased expression of lncRNA NEAT1 and decreased expression of miR-23b-3p in SNFH. (a) The expression of lncRNA NEAT1 in tissues and cells; (b) the expression of miR-23b-3p in tissues and cells; (c) ROC curve suggested that lncRNA NEAT1 was valuable in the diagnosis of SNFH; (d) lncRNA NEAT1 was negatively correlated with miR-23b-3p. Compared with the control group, ^*^ *P* < 0.05. SNFH: steroid-induced necrosis of femoral head.

qRT-PCR was used to detect the expression of lncRNA NEAT1 and miR-23b-3p in tissues. The results showed that compared to the control group, the expression of lncRNA NEAT1 in SNFH tissues and the corresponding hBMSCs was increased (Figure 3a, both *P* < 0.05), and the expression of miR-23b-3p was decreased (Figure 3b, both *P* < 0.05). The expression of lncRNA NEAT1 was negatively correlated with miR-23b-3p (Figure 3d, *P* < 0.05). ROC curve showed that lncRNA NEAT1 had a diagnostic value for SNFH (Figure 3c, *P* < 0.05). The above results indicate that lncRNA NEAT1 may be a potential target for the treatment of SNFH.

### 3.4 lncRNA NEAT1 knockdown can promote the proliferation and osteogenesis of hBMSCs

**Figure 4:**
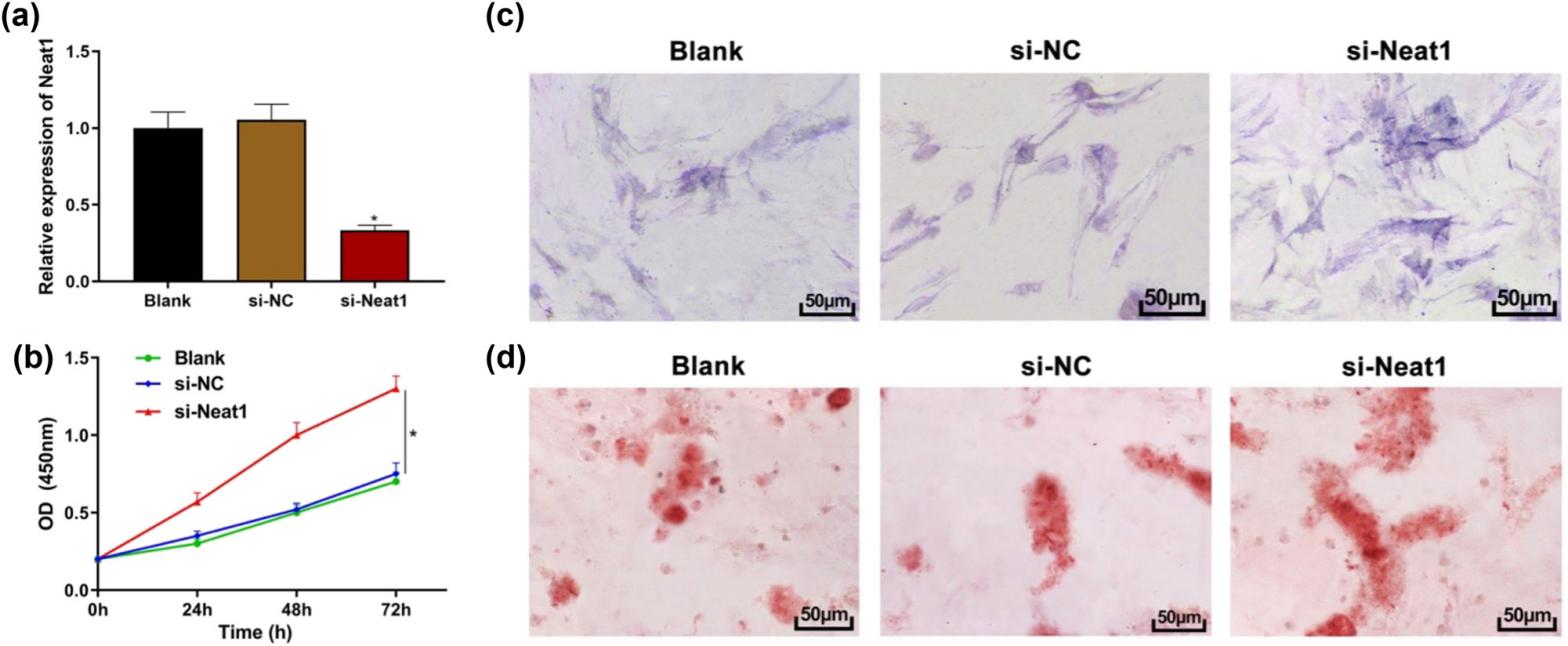
lncRNA NEAT1 knockdown can promote the proliferation and osteogenesis of hBMSCs. (a) qRT-PCR to detect the transfection efficiency of lncRNA NEAT1 knockdown; (b) CCK8 assay to detect cell proliferation; (c) ALP staining (200×); (d) ARS staining (200×). Compared with the si-NC group, ^*^ *P* < 0.05. ALP: alkaline phosphatase. ARS: alizarin red staining.

To further study the effect of lncRNA NEAT1 on GC-induced hBMSCs, hBMSCs were transfected with siRNAs for the knockdown of lncRNA NEAT1, and the following experiments were carried out. qRT-PCR showed that lncRNA NEAT1 knockdown significantly reduced the expression of lncRNA NEAT1 in hBMSCs (Figure 4a, *P* < 0.05). CCK8 assay was carried out to investigate the effect of lncRNA NEAT1 on the proliferation of hBMSCs. The results showed that when compared with the control group, knockdown of lncRNA NEAT1 could rescue hBMSCs apoptosis caused by GC stimulation and promote the proliferation of hBMSCs (Figure 4b, *P* < 0.05). Next, we conducted osteoinduction to study the effect of lncRNA NEAT1 on osteogenesis. After 21 days of osteoinduction, ALP and ARS staining showed that compared to the control group, ALP staining after knockdown of lncRNA NEAT1 was more apparent (Figure 4c), and calcium nodules after knockdown of lncRNA NEAT1 increased in ARS staining (Figure 4d), indicating that silencing lncRNA NEAT1 could promote the osteogenesis of hBMSCs. The above results indicate that the knockdown of lncRNA NEAT1 can promote the proliferation and osteogenesis of hBMSCs.

### 3.5 Overexpression of miR-23b-3p can partially counteract the effect of lncRNA NEAT1 on hBMSCs

**Figure 5:**
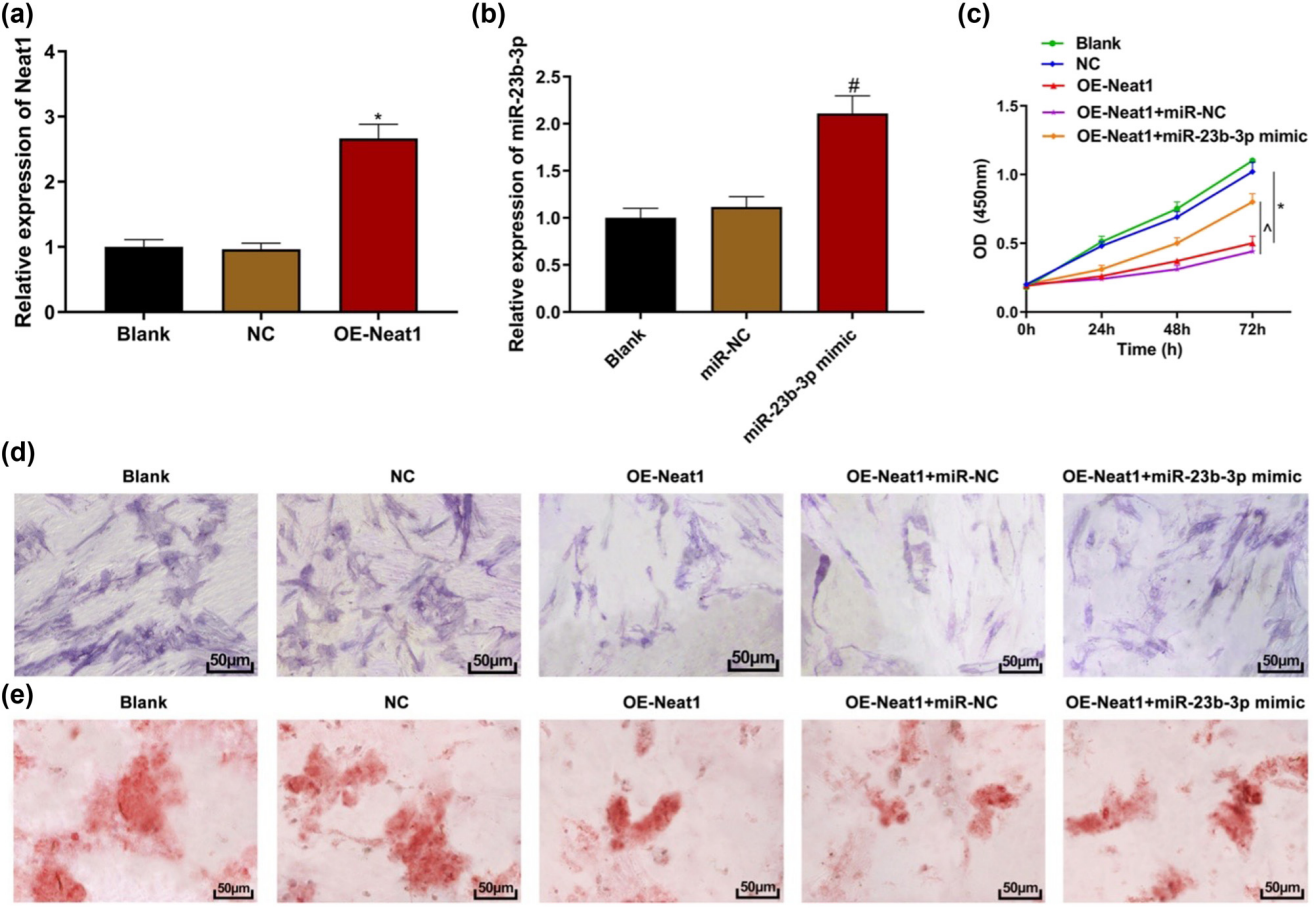
Overexpression of miR-23b-3p can partially counteract the effect of lncRNA NEAT1 on hBMSCs. (a) and (b) qRT-PCR to detect the transfection efficiency of lncRNA NEAT1 and miR-23b-3p overexpression plasmids; (c) CCK8 assay to detect cell proliferation; (d) ALP staining (200×); (e) ARS staining (200×). Compared with the NC group, ^*^ *P* < 0.05; compared with the miR-NC group, ^#^ *P* < 0.05; compared with the OE-NEAT1 + miR-NC group, ^^^ *P* < 0.05. NC: negative control. ALP: alkaline phosphatase. ARS: alizarin red staining.

hBMSCs were co-transfected with lncRNA NEAT1 and miR-23b-3p overexpression plasmids to investigate the relationship between lncRNA NEAT1 and miR-23b-3p. qRT-PCR showed that when compared with the negative control group, transfection with lncRNA NEAT1 plasmid and miR-23b-3p plasmid increased the expression of lncRNA NEAT1 and miR-23b-3p, respectively (Figure 5a and b, both *P* < 0.05). CCK8 assay showed that when compared with the control group, the overexpression of lncRNA NEAT1 could inhibit the proliferation of hBMSCs. When miR-23b-3p was upregulated, the effect of lncRNA NEAT1 overexpression on hBMSCs was partially counteracted (Figure 5c, both *P* > 0.05). After 21 days of osteoinduction, the overexpression of lncRNA NEAT1 resulted in a lighter ALP staining, while the addition of miR-23b-3p mimic increased the ALP staining (Figure 5d). The ARS results showed what overexpression of lncRNA NEAT1 resulted in fewer calcium nodules, while the addition of miR-23b-3p mimic increased the calcium nodules (Figure 5e). The above results indicate that overexpression of miR-23b-3p can partially counteract the effect of lncRNA NEAT1 on hBMSCs.

### 3.6 CYP1A2 is a target of miR-23b-3p

**Figure 6:**
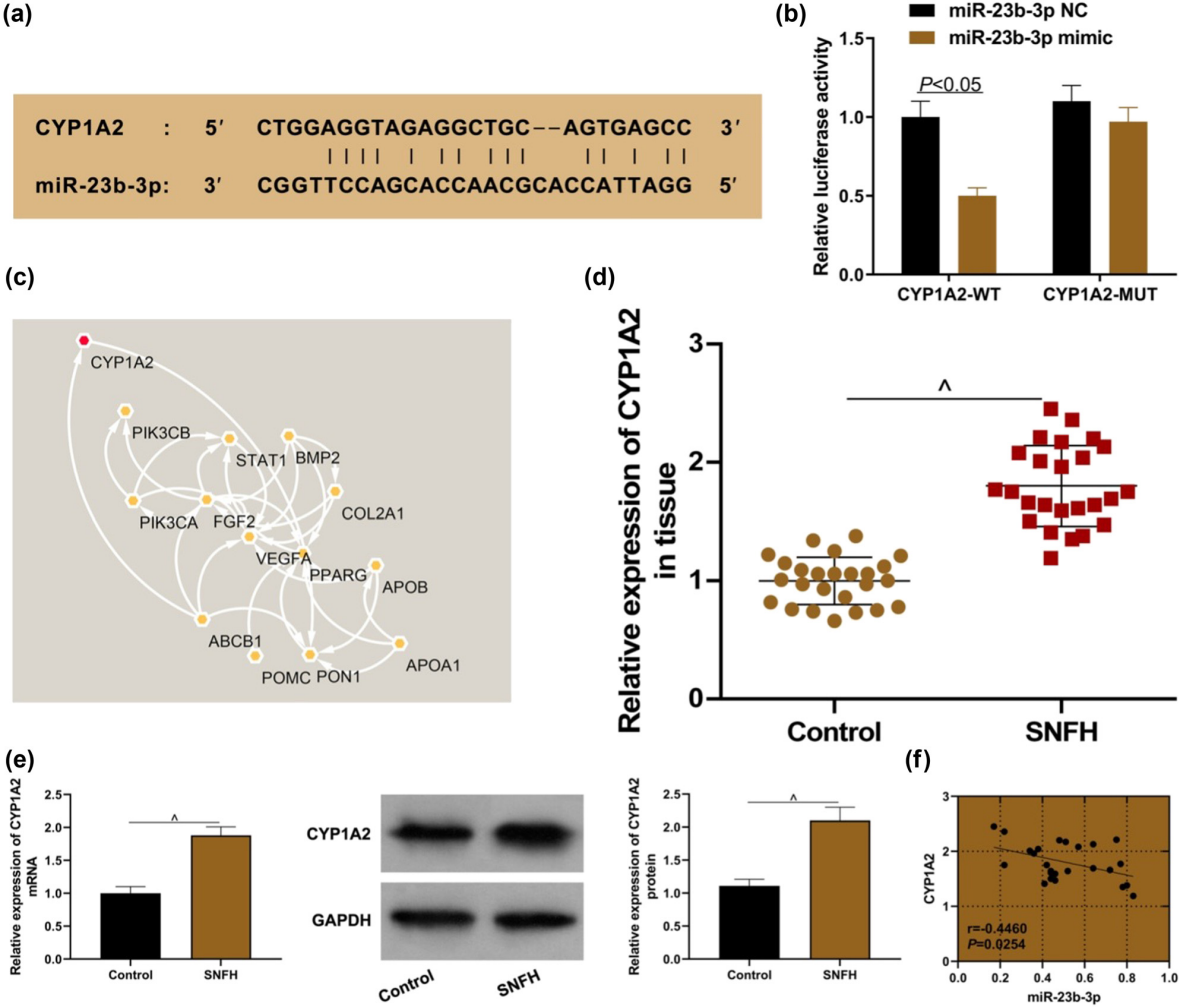
CYP1A2 is a target of miR-23b-3p. (a) RNA22 database predicted that CYP1A2 and miR-23b-3p had a binding site; (b) dual luciferase reporter assay indicated that there was a targeting relationship between CYP1A2 and miR-23b-3p; (c) String database predicted that CYP1A2 was associated with risk genes for SNFH; (d) CYP1A2 expression in SNFH tissues; (e) CYP1A2 expression in hBMSCs; (f) CYP1A2 and miR-23b-3p were negatively correlated. Compared with the control group, ^^^ *P* < 0.05. SNFH: steroid-induced necrosis of femoral head; hBMSCs: human bone marrow mesenchymal stem cells.

The RNA22 database showed that CYP1A2 and miR-23b-3p had a binding site (Figure 6a). Dual-luciferase reporter assay was designed to confirm the targeting relationship between CYP1A2 and miR-23b-3p. The results showed that compared to the CYP1A2-WT + miR-23b-3p NC group, the luciferase activity of the CYP1A2-WT + miR-23b-3p mimic group was significantly decreased (Figure 6b, *P* < 0.05). However, there was no significant difference in the luciferase activity between CYP1A2-MUT + miR-23b-3p mimic and its corresponding control group (Figure 6b, *P* > 0.05). The String database showed that CYP1A2 was associated with risk genes for SNFH (Figure 6c). qRT-PCR results showed that when compared with the control group, the expression of CYP1A2 was upregulated in SNFH (Figure 6d, *P* < 0.05), and the mRNA and protein expression of CYP1A2 were upregulated in corresponding hBMSCs (Figure 6e, *P* < 0.05). There was a negative correlation between the expression of CYP1A2 and miR-23b-3p in SNFH tissues (Figure 6f, *P* < 0.05). The above results indicate that CYP1A2 is regulated by miR-23b-3p and is highly expressed in SNFH.

### 3.7 Overexpression of CYP1A2 can partially rescue the effect of miR-23b-3p overexpression on hBMSCs

**Figure 7:**
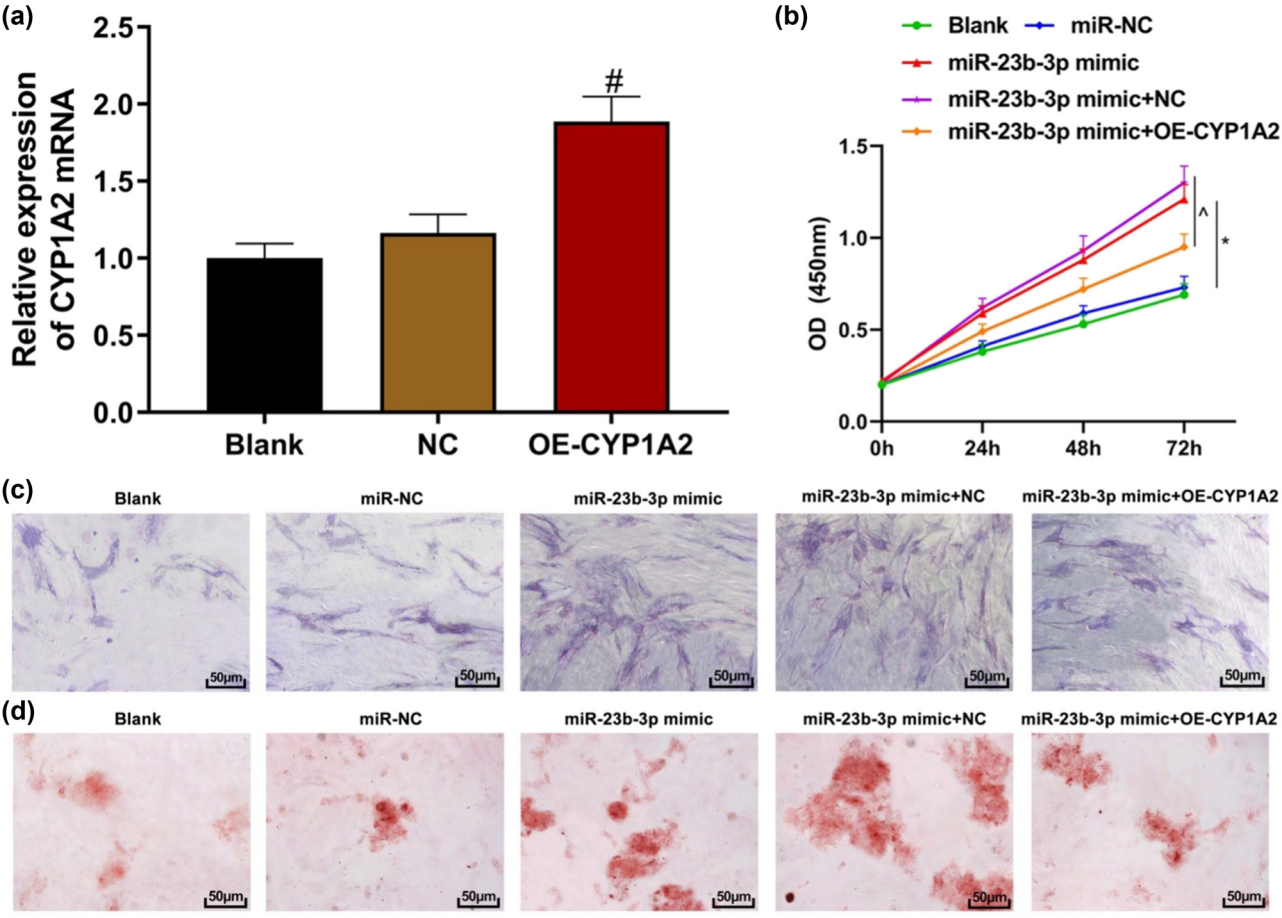
Overexpression of CYP1A2 can partially rescue the effect of miR-23b-3p overexpression on hBMSCs. (a) qRT-PCR to detect the transfection efficiency of CYP1A2 overexpression plasmid; (b) CCK8 assay to detect cell proliferation; (c) ALP staining (200×); (d) ARS staining (200×). Compared with the NC group, ^#^ *P* < 0.05; compared with the miR-NC group, ^*^ *P* < 0.05; compared with the miR-23b-3p mimic + NC group, ^^^ *P* < 0.05. ALP: alkaline phosphatase; ARS: alizarin red staining; hBMSCs: human bone marrow mesenchymal stem cells.

The targeted regulatory relationship between CYP1A2 and miR-23b-3p had been verified. Next, hBMSCs were co-transfected with CYP1A2 and miR-23B-3p overexpression plasmids to discuss the functional relationship between CYP1A2 and miR-23b-3p. qRT-PCR showed that transfection with CYP1A2 overexpression plasmids significantly increased the expression of CYP1A2 mRNA in hBMSCs (Figure 7a, *P* < 0.05). CCK8 assay showed that when compared with the miR-NC group, the proliferation of hBMSCs was promoted in the miR-23b-3p mimic group. Compared to the miR-23b-3p mimic + NC group, the proliferation of hBMSCs in miR-23b-3p mimic + OE-CYP1A2 group was inhibited (Figure 7b, both *P* < 0.05). ALP staining results showed that the staining miR-23b-3p mimic group was heavier than that of the miR-NC group, and the staining of the miR-23b-3p mimic + OE-CYP1A2 group was lighter than that of the mir-23b-3p mimic + NC group (Figure 7c). ARS staining results showed that compared to the miR-NC group, there were more calcium nodules in the miR-23b-3p mimic group. Compared to the miR-23b-3p mimic + NC group, the miR-23b-3p mimic + OE-CYP1A2 group had fewer calcium nodules (Figure 7d). The above results indicate that overexpression of miR-23b-3p can promote the proliferation and osteogenic differentiation of hBMSCs, and overexpression of CYP1A2 can partially rescue the above effect.

## 4 Discussion

Excessive intake of GCs is considered to be the leading cause of SNFH [18]. In recent years, the study on SNFH has focused on lncRNA and miRNA, which have attracted much attention because of their different biological functions for bone remodeling [19]. The role of lncRNA NEAT1 in SNFH has not been discussed before, so this study is the first to explore the function of lncRNA NEAT1 in SNFH bone remodeling. In this study, we first intervened hBMSCs with GCs to construct an *in vitro* injury model, and then we intervened the gene expression and observed the effect on the model. Bone remodeling is a very complex process. Both local and systemic cytokines are at play to form a complex remodeling network [20]. GCs affect bone remodeling by many mechanisms, participating in any stage of the remodeling cycle [21]. GCs can directly act on two kinds of cells that are very important in bone remodeling: osteoblasts and osteoclasts. A study has shown that small doses of GCs can promote osteogenic differentiation, while high doses of GCs can inhibit the formation of osteoblasts. Similarly, high doses of GCs can promote the proliferation of osteoclasts, increase bone resorption, cause bone loss and affect bone remodeling [22]. BMSCs are a subgroup of cells with multi-differentiation potential that can differentiate into bone, cartilage, fat, myoblasts and nerves. The process of bone formation is inseparable from the role of BMSCs [23]. In this study, we found that lncRNA NEAT1 was highly expressed in SNFH tissues. Knockdown of lncRNA NEAT1 could promote the proliferation and osteogenic differentiation of hBMSCs. In addition, the diagnostic value of lncRNA NEAT1 in SNFH was analyzed by the ROC curve, and the result showed that the value of area under curve was above 0.85. It suggested that lncRNA NEAT1 is a potential biomarker of SNFH, which has a certain value for the diagnosis and treatment of SNFH. lncRNA has recently been a hotspot in many diseases, such as cancer, heart diseases and diabetes [24,25,26]. These studies show that lncRNA can be used as a specific marker in the body and plays an important role in the pathological process of diseases. In this study, lncRNA NEAT1, as mentioned before, is also involved in the pathological development of diseases. A study found that lncRNA NEAT1 could be used as a biomarker of multiple myeloma, and myeloma cells could inhibit the differentiation of BMSCs into osteoblasts [27]. Overexpression of NEAT1 has also been shown to promote osteoclast formation [12]. lncRNA can be combined with miRNA as a competitive endogenous RNA to play a role [28]. The Starbase website was used to predict the miRNA with specific binding sites to lncRNA NEAT1. Finally, we focus on miR-23b-3p. A previous study found that miR-23b-3p was downregulated in SNFH, and overexpression of miR-23b-3p could relieve symptoms of SNFH rats, reduce pro-inflammatory cytokines and blood lipids and promote bone integrity [15]. Therefore, this study speculates that lncRNA NEAT1 may participate in the progress of SNFH through interaction with miR-23b-3p. This study further confirmed that miR-23b-3p is the downstream target gene of lncRNA NEAT1. The expression of miR-23b-3p was downregulated in SNFH, and miR-23b-3p has a negative correlation with lncRNA Neat1. Upregulation of miR-23b-3p could partially counteract the malignant effect of lncRNA NEAT1 on hBMSCs. To find possible targets of miR-23b-3p, we predict the target of miR-23b-3p using the RNA22 website and finally focus on CYP1A2 to be investigated in the study. A previous study found that the expression of CYP1A2 was upregulated in the tissues of patients with SNFH [16]. Through the online prediction website of STRING, we found an association between CYP1A2 and the risk gene of SNFH. The dual-luciferase reporter assay confirmed that CYP1A2 played a role as a downstream target gene of miR-23b-3p. Finally, we found that overexpression of miR-23b-3p could promote the proliferation and osteogenic differentiation of hBMSCs, and this effect was partially rescued by overexpression of CYP1A2. However, this study also has some limitations. We only discussed the effect of lncRNA NEAT1 *in vitro*. However, its effect *in vivo* is still unknown, which will be a direction for our future research.

In conclusion, the results of this study show that lncRNA NEAT1 upregulates the expression of CYP1A2 by adsorbing miR-23b-3p to inhibit the proliferation and osteogenic differentiation of BMSCs. lncRNA NEAT1 is likely to be a potential target for the treatment of SNFH.

## References

[1] Tian L, Sun S, Li W, Yuan L, Wang X. Down-regulated microRNA-141 facilitates osteoblast activity and inhibits osteoclast activity to ameliorate osteonecrosis of the femoral head via upregulating TGF-β2. Cell Cycle (Georgetown, Tex). 2020;19:772–86. 10.1080/15384101.2020.1731053 PMC7145325 32089067

[2] Kuang MJ, Xing F, Wang D, Sun L, Ma JX, Ma XL. CircUSP45 inhibited osteogenesis in glucocorticoid-induced osteonecrosis of femoral head by sponging miR-127-5p through PTEN/AKT signal pathway: experimental studies. Biochem Biophys Res Commun. 2019;509:255–61. 10.1016/j.bbrc.2018.12.116 30579595

[3] Sun Z, Wu F, Yang Y, Liu F, Mo F, Chen J, et al. MiR-144-3p inhibits BMSC proliferation and Osteogenic differentiation via targeting FZD4 in steroid-associated Osteonecrosis. Curr Pharm Des. 2019;25:4806–12. 10.2174/1381612825666190930094019 31566128

[4] Xu J, Gong H, Lu S, Deasey MJ, Cui Q. Animal models of steroid-induced osteonecrosis of the femoral head-a comprehensive research review up to 2018. Int Orthop. 2018;42:1729–37. 10.1007/s00264-018-3956-1 29705870

[5] Al-Hrout A, Chaiboonchoe A, Khraiwesh B, Murali C, Baig B, El-Awady R, et al. Safranal induces DNA double-strand breakage and ER-stress-mediated cell death in hepatocellular carcinoma cells. Sci Rep. 2018;8:16951. 10.1038/s41598-018-34855-0 PMC6240095 30446676

[6] Hamza A, Fikry E, Abdallah W, Amin A. Mechanistic insights into the augmented effect of bone marrow mesenchymal stem cells and thiazolidinediones in streptozotocin-nicotinamide induced diabetic rats. Sci Rep. 2018;8:9827. 10.1038/s41598-018-28029-1 PMC6026169 29959408

[7] Wang Q, Yang Q, Chen G, Du Z, Ren M, Wang A, et al. lncRNA expression profiling of BMSCs in osteonecrosis of the femoral head associated with increased adipogenic and decreased osteogenic differentiation. Sci Rep. 2018;8:9127. 10.1038/s41598-018-27501-2 PMC6002551 29904151

[8] Xiang S, Li Z, Weng X. The role of lncRNA RP11-154D6 in steroid-induced osteonecrosis of the femoral head through BMSC regulation. J Cell Biochem. 2019;120:18435–45. 10.1002/jcb.29161 31190361

[9] Cheng H, Malhotra A. Evaluation of potential of long noncoding RNA NEAT1 in colorectal cancer. J Env Pathol Toxicol Oncol. 2020;39:101–11. 10.1615/JEnvironPatholToxicolOncol.2020032508 32749120

[10] Xue F, Cheng Y, Xu L, Tian C, Jiao H, Wang R, et al. lncRNA NEAT1/miR-129/Bcl-2 signaling axis contributes to HDAC inhibitor tolerance in nasopharyngeal cancer. Aging. 2020;12:14174–88. 10.18632/aging.103427 PMC7425502 32692721

[11] Li C, Liu YF, Huang C, Chen YX, Xu CY, Chen Y. Long non-coding RNA NEAT1 sponges miR-129 to modulate renal fibrosis by regulation of collagen type I. Am J Physiol Ren Physiol. 2020;319:93–105. 10.1152/ajprenal.00552.2019 32475133

[12] Zhang Y, Chen XF, Li J, He F, Li X, Guo Y. lncRNA Neat1 stimulates Osteoclastogenesis via sponging miR-7. J Bone Min Res. 2020;35:1772–81. 10.1002/jbmr.4039 32353178

[13] Ji S, Wang S, Zhao X, Lv L. Long non-coding RNA NEAT1 regulates the development of osteosarcoma through sponging miR-34a-5p to mediate HOXA13 expression as a competitive endogenous RNA. Mol Genet Genomic Med. 2019;7:673. 10.1002/mgg3.673 PMC6565592 31044561

[14] Zhao CH, Bai XF, Hu XH. Knockdown of lncRNA XIST inhibits hypoxia-induced glycolysis, migration and invasion through regulating miR-381-3p/NEK5 axis in nasopharyngeal carcinoma. Eur Rev Med Pharmacol Sci. 2020;24:2505–17. 10.26355/eurrev_202003_20518 32196601

[15] Liu Y, Zong Y, Shan H, Lin Y, Xia W, Wang N, et al. microRNA-23b-3p participates in steroid-induced osteonecrosis of the femoral head by suppressing ZNF667 expression. Steroids. 2020;163:108709. 10.1016/j.steroids.2020.108709 32730776

[16] Wei JH, Luo QQ, Tang YJ, Chen JX, Huang CL, Lu DG, et al. Upregulation of microRNA-320 decreases the risk of developing steroid-induced avascular necrosis of femoral head by inhibiting CYP1A2 both in vivo and in vitro. Gene. 2018;660:136–44. 10.1016/j.gene.2018.03.045 29551500

[17] Wang X, Du Z, Liu X, Song Y, Zhang G, Wang Z, et al. Expression of CD44 standard form and variant isoforms in human bone marrow stromal cells. Saudi Pharm J. 2017;25:488–91. 10.1016/j.jsps.2017.04.011 PMC5447408 28579880

[18] Xie Y, Hu JZ, Shi ZY. MiR-181d promotes steroid-induced osteonecrosis of the femoral head by targeting SMAD3 to inhibit osteogenic differentiation of hBMSCs. Eur Rev Med Pharmacol Sci. 2018;22:4053–62. 10.26355/eurrev_201807_15393 30024590

[19] Wei B, Wei W, Zhao B, Guo X, Liu S. Long non-coding RNA HOTAIR inhibits miR-17-5p to regulate osteogenic differentiation and proliferation in non-traumatic osteonecrosis of femoral head. PLoS One. 2017;12:0169097. 10.1371/journal.pone.0169097 PMC5312925 28207735

[20] Deshet-Unger N, Kolomansky A, Ben-Califa N, Hiram-Bab S, Gilboa D, Liron T, et al. Erythropoietin receptor in B cells plays a role in bone remodeling in mice. Theranostics. 2020;10:8744–56. 10.7150/thno.45845 PMC7392011 32754275

[21] Chiodini I, Merlotti D, Falchetti A, Gennari L. Treatment options for glucocorticoid-induced osteoporosis. Expert Opin Pharmacother. 2020;21:721–32. 10.1080/14656566.2020.1721467 32004105

[22] Wang XB, Li PB, Guo SF, Yang QS, Chen ZX, Wang D, et al. circRNA_0006393 promotes osteogenesis in glucocorticoid‑induced osteoporosis by sponging miR‑145‑5p and upregulating FOXO1. Mol Med Rep. 2019;20:2851–8. 10.3892/mmr.2019.10497 31322188

[23] Zhang G, Li H, Zhao W, Li M, Tian L, Ju W, et al. miR-205 regulates bone turnover in elderly female patients with type 2 diabetes mellitus through targeted inhibition of Runx2. Exp Ther Med. 2020;20:1557–65. 10.3892/etm.2020.8867 PMC7388399 32742387

[24] Xiao Z, Liu Y, Zhao J, Li L, Hu L, Lu Q, et al. Long non-coding RNA LINC01123 promotes the proliferation and invasion of hepatocellular carcinoma cells by modulating the miR-34a-5p/TUFT1 axis. Int J Biol Sci. 2020;16:2296–305. 10.7150/ijbs.45457 PMC7378647 32760198

[25] Zhang G, Dou L, Chen Y. Association of long-chain non-coding RNA MHRT gene single nucleotide polymorphism with risk and prognosis of chronic heart failure. Med. 2020;99:19703. 10.1097/MD.0000000000019703 PMC7373586 32702806

[26] Fan W, Wen X, Zheng J, Wang K, Qiu H, Zhang J, et al. LINC00162 participates in the pathogenesis of diabetic nephropathy via modulating the miR-383/HDAC9 signalling pathway. Artif Cell Nanomed Biotechnol. 2020;48:1047–54. 10.1080/21691401.2020.1773487 32677473

[27] Yu H, Peng S, Chen X, Han S, Luo J. Long non-coding RNA NEAT1 serves as a novel biomarker for treatment response and survival profiles via microRNA-125a in multiple myeloma. J Clin Lab Anal. 2020;34:23399. 10.1002/jcla.23399 PMC7521229 32608537

[28] Fu D, Yang S, Lu J, Lian H, Qin K. lncRNA NORAD promotes bone marrow stem cell differentiation and proliferation by targeting miR-26a-5p in steroid-induced osteonecrosis of the femoral head. Stem Cell Res Ther. 2021;12:18. 10.1186/s13287-020-02075-x PMC7792292 33413642

